# Deficiency in Calcium-Binding Protein S100A4 Impairs the Adjuvant Action of Cholera Toxin

**DOI:** 10.3389/fimmu.2017.01119

**Published:** 2017-09-11

**Authors:** Jia-Bin Sun, Jan Holmgren, Maximilian Larena, Manuela Terrinoni, Yu Fang, Anne R. Bresnick, Zou Xiang

**Affiliations:** ^1^Department of Microbiology and Immunology, Institute of Biomedicine, University of Gothenburg Vaccine Research Institute (GUVAX), Sahlgrenska Academy at University of Gothenburg, Gothenburg, Sweden; ^2^Department of Microbiology and Immunology, Clinical Research Center, Affiliated Hospital of Guizhou Medical University, Guiyang, China; ^3^Department of Biochemistry, Albert Einstein College of Medicine, Bronx, NY, United States; ^4^Faculty of Health and Social Sciences, Department of Health Technology and Informatics, The Hong Kong Polytechnic University, Hong Kong, Hong Kong

**Keywords:** adjuvant, cholera toxin, dendritic cells, germinal center, S100A4

## Abstract

The calcium-binding protein S100A4 has been described to promote pathological inflammation in experimental autoimmune and inflammatory disorders and in allergy and to contribute to antigen presentation and antibody response after parenteral immunization with an alum-adjuvanted antigen. In this study, we extend these findings by demonstrating that mice lacking S100A4 have a defective humoral and cellular immune response to mucosal (sublingual) immunization with a model protein antigen [ovalbumin (OVA)] given together with the strong mucosal adjuvant cholera toxin (CT), and that this impairment is due to defective adjuvant-stimulated antigen presentation by antigen-presenting cells. In comparison to wild-type (WT) mice, mice genetically lacking S100A4 had reduced humoral and cellular immune responses after immunization with OVA plus CT, including a complete lack of detectable germinal center reaction. Further, when stimulated *in vitro* with OVA plus CT, S100A4^−/−^ dendritic cells (DCs) showed impaired responses in several CT-stimulated immune regulatory molecules including the co-stimulatory molecule CD86, inflammasome-associated caspase-1 and IL-1β. Coculture of OVA-specific OT-II T cells with S100A4^−/−^ DCs that had been pulse incubated with OVA plus CT resulted in impaired OT-II T cell proliferation and reduced production of Th1, Th2, and Th17 cytokines compared to similar cocultures with WT DCs. In accordance with these findings, transfection of WT DCs with S100A4-targeting small interfering RNA (siRNA) but not mock-siRNA resulted in significant reductions in the expression of caspase-1 and IL-1β as well as CD86 in response to CT. Importantly, also engraftment of WT DCs into S100A4^−/−^ mice effectively restored the immune response to immunization in the recipients. In conclusion, our results demonstrate that deficiency in S100A4 has a strong impact on the development of both humoral and cellular immunity after mucosal immunization using CT as adjuvant.

## Introduction

S100A4 is one of more than 20 structurally related calcium-binding proteins of the S100 protein family, which have intracellular as well as extracellular functions (1, 2). S100A4 is expressed in many normal cells, including fibroblasts, endothelial cells, smooth muscle cells, lymphocytes, neutrophils, and macrophages (3–5). Calcium binding to S100A4 induces a conformational rearrangement that allows S100A4 to interact with a number of cellular targets and exert biological effects (2). S100A4 has been found to regulate a diverse range of cellular processes such as cell growth and survival, differentiation, and motility (6). Clinically oriented research on S100A4 has been largely focused on its cancer metastasis-promoting properties (3, 7, 8), and its role in promoting pathological inflammation in rheumatoid arthritis (9, 10), cardiovascular disease (11), fibrotic diseases (12, 13), and experimental autoimmune encephalomyelitis (14). Loss of S100A4 has been shown to impair macrophage recruitment and chemotactic motility to sites of inflammation *in vivo* (4). Recently, we also identified a central role for S100A4 in allergy (15).

The receptor for advanced glycation end products (RAGE), the epidermal growth factor receptor, and toll-like receptors (TLRs) have been identified as potential extracellular receptors for S100 proteins (2, 16). In various cancer models, S100A4 has been demonstrated to signal either through RAGE or other as yet undefined receptors leading to mobilization of NF-κB (17–21). S100A4 signaling *via* TLR4 and activation of NF-κB are required for the production of proinflammatory cytokines by mononuclear cells in the pathogenesis of rheumatoid arthritis (10).

In connection with our studies on the role of S100A4 in allergic pathology (15), we noted that S100A4-deficient mice had reduced antibody responses to parenteral immunization with a model protein antigen [ovalbumin (OVA)] adjuvanted with alum, and that S100A4^−/−^ dendritic cells (DCs) poorly activated T cells *in vitro* (15). In this study, we examined the role of S100A4 in the development of humoral and cellular immune responses, including germinal center reactions after mucosal immunization with OVA together with the strong mucosal adjuvant, cholera toxin (CT). Our results indicate that S100A4 expression in antigen-presenting cells (APC) is required for effective CT-adjuvant-dependent mucosal immunization and is critical for germinal center formation. This is linked to deficient antigen presentation in S100A4^−/−^ mice in response to immunization, as engraftment of wild-type (WT) DCs to S100A4^−/−^ mice before immunization fully restored immune responsiveness *in vivo*. Consistent with this, S100A4^−/−^ DCs were found to have reduced antigen-presenting capacity that is associated with defects in the expression of adjuvant-induced inflammasome-associated caspase-1 and IL-1β, and co-stimulatory molecules. Our results demonstrate a strong effect of S100A4-deficiency in the adjuvant action of CT and, as discussed, possibly also of other adjuvants.

## Materials and Methods

### Experimental Animals

S100A4^−/−^ mice on a C57BL/6 background were generated as described previously (4) and were bred in-house at the MIVAC breeding unit of the experimental animal facility of the University of Gothenburg. OT-IIxLy5.1 mice on the C57BL/6 background whose T cells have a transgenic T cell receptor specific for the 323–339 peptide of OVA (OVA_323–339_) were obtained from the MIVAC breeding unit. Female mice were 6–8 weeks old at the start of all experiments. All mice, including C57BL/6 WT control mice (purchased from B&K Universal AB, Stockholm, Sweden), were housed together under specific pathogen-free conditions at the experimental animal facility for at least 2 weeks before and for the duration of the experiments. The studies were approved by the University of Gothenburg Ethical Committee for Animal Experimentation.

### Immunization and Collection of Specimens

Ovalbumin (grade VII) was purchased from Sigma (St. Louis, MO, USA). For sublingual (s.l.) immunization, S100A4^+/+^ and S100A4^−/−^ mice were treated twice with 200 µg OVA mixed with or without 5 µg CT in a 10-µl droplet placed under the tongue at an interval of 10 days unless otherwise stated. Ten days after the last s.l. treatment, serum, cervical lymph nodes (CLNs), and spleens were collected and analyzed. Alternatively, spleens and CLNs were removed 3 days after the second immunization to assess germinal center formation. In some experiments, CLNs were removed 2 days after a single immunization to assess early-stage responses.

### Isolation or Generation of DCs and T Cells

Minced spleens and lymph nodes were digested in 25 µg/ml liberase (Roche Applied Science, Mannheim, Germany) and 400 U/ml DNase I (Roche) at 37°C for 30 min followed by filtration through a 40-µm nylon net. CD11c^+^ DCs were then isolated by positive selection using MACS microbeads coated with antibodies against mouse CD11c (Miltenyi Biotec, Auburn, CA, USA) according to the manufacturer’s instructions. The purity of the isolated DCs was >90%. In some experiments, bone marrow-derived DCs (BMDCs) were generated by culturing bone marrow (BM) cells in the presence of 200 ng/ml Flt3-L for 9 days as previously described (22).

CD4^+^ T cells were purified from spleens and lymph nodes initially as described earlier followed by positive selection using MACS microbeads labeled with an anti-CD4 antibody (Miltenyi) in accordance with the manufacturer’s recommended protocol.

### T Cell Proliferation and Cytokine Assays

Splenocytes (2 × 10^5^/well) were cultured for 3 days in 96-well plates in the presence of 20 µg/ml OVA in 200 µl IMDM supplemented with 10% fetal calf serum, 1% l-glutamine, 1% gentamicin, and 50 µM mercaptoethanol. [^3^H]-thymidine (1 μCi/well) was added during the last 8 h, and its incorporation was measured as a marker of T cell proliferation.

In some proliferation assays, BMDCs were pulsed with 20 µg/ml OVA together with 0.1 µg/ml CT for 2 h. This was followed by rigorous cell washing using three rounds of centrifugation. Next, the DCs (2 × 10^4^/well) were cocultured with freshly isolated OT-II T cells (2 × 10^5^/well) for 3 days. Supernatants were collected prior to the addition of [^3^H]-thymidine for cytokine assays. Production of IL-1β, IFN-γ, and IL-17 in the culture supernatants was measured using the Meso Scale Discovery (MSD) method according to the manufacturer’s instructions (MSD, Rockville, MD, USA).

### Adoptive Transfer Experiment

Freshly isolated CD11c^+^ DCs were adoptively transferred by an intravenous injection of 2 × 10^6^ cells in 200 µl PBS into the tail vein, and one day later the recipient mice were s.l. immunized as described earlier.

### DC Stimulation by CT

Freshly prepared BMDCs were pulsed *in vitro* with 20 µg/ml OVA in the presence or absence of 100 ng/ml CT overnight at 37°C in 5% CO_2_ followed by rigorous washing and resuspension in medium.

### Flow Cytometric Staining and Analyses

For staining of surface markers, cells were incubated with antibodies to mouse CD4, CD25, CD103, CD69, CD138, B220, CD11c, CD19, CD80, CD62L, CD86, ICOS, CXCR5, PD-1, CD38, GL7, and CD45.1 (BD Pharmingen, San Jose, CA, USA or eBioscience, San Diego, CA, USA). For detection of intracellular Foxp3, cells were fixed and permeabilized with Cytofix/Cytoperm solution (eBioscience) according to the manufacturer’s recommended protocol, followed by incubation with FcγR block for 15 min and an anti-Foxp3 antibody (Clone FLK-16; Nordic Biosite, Taby, Sweden) at 4°C for 30 min.

For measurement of caspase-1, DCs were incubated overnight either in the presence or absence of CT (100 ng/ml). Cells were stained for the surface markers CD11c and MHC class II, together with CD80, CD86, and 7-AAD. This was followed by staining with FAM-YVAD fmk for 1 h at 37°C using the caspase-1 FLICA kit from ImmunoChemistry Technologies (Bloomington, MN, USA) according to the kit instructions. For measuring intracellular IL-1β expression, cells were pre-incubated in the presence or absence of 100 ng/ml CT for 2 h at 37°C. After washing, cells were cultured for 16 h and then stained for surface markers as described earlier, followed by intracellular staining of a preform of-IL-1β (eBiosciences, San Diego, CA, USA). Cells were analyzed by an LSR II flow cytometer (BD). Data were analyzed by FlowJo software (Tree Star, Ashland, OR, USA).

### Confocal Microscopy

Three days after the second s.l. immunization, spleens were removed and embedded in Tissue-Tek OCT compound (Sakura, Zoeterwoude, The Netherlands). For some analyses, tissues were immediately frozen in liquid nitrogen and stored at −70°C. 7-μm-thick frozen tissue sections were prepared and then fixed in acetone, air-dried, and rinsed in PBS. Spleen cryosections were stained with anti-B220, GL7, and Ki67 (anti-B220-Biotin, BD #553086 followed by Streptavidin Alexa Fluor 594, Invitrogen #S11227; GL7-eFluor 660, eBioscience #50-5902-82; Ki67-V450, BD #561281). Slides were mounted with Dako fluorescent mounting medium #S3023 (Dako, Glostrup, Denmark). Confocal images were acquired using an inverted LSM 700 Axio Observer.Z1 under Plan-Apochromat 20×/1.4 DIC immersion objective (Carl Zeiss, Jena, Germany).

### PCR Array

Bone marrow-derived DCs (3 × 10^6^) were left untreated or treated with 1 µg/ml CT for 16 h at 37°C in 5% CO_2_. Total RNA was extracted using the RNeasy Mini-Kit (Qiagen, Hilden, Germany), quality checked with 2200 Tapestation (Agilent Technologies, Santa Clara, CA, USA), and cDNA generated from 1 µg of total RNA using QuantiTect Reverse Transcription Kit (Qiagen). Quantitative real-time PCR was performed using a customized RT2 Profiler PCR Array System (Qiagen) according to the manufacturer’s instructions. Data were normalized to PPIA gene expression and analyzed using a web-based software package for the PCR array system (Qiagen).

### S100A4 Gene Silencing by siRNA in DCs

S100A4-targeting small interfering RNAs (siRNA) and nonsense siRNA were purchased from Life Technologies (Carlsbad, CA, USA). For transfections, cells were seeded at a density of 5 × 10^5^ cells/well in a 48-well-plate with Opti-MEM reduced serum-free medium (Gibco Life Technologies, Carlsbad, CA, USA). Appropriately diluted S100A4 or control nonsense siRNA (150 µl) was mixed with Lipofectamine-RNAiMAX reagent (150 µl) in Opti-MEM medium and allowed to form complexes at room temperature for 5 min. Next, each well of DCs was treated with the siRNA complex media (final concentration of 25 pM siRNA). After 4 h, cells were treated with normal serum-supplemented medium containing Flt3-L. After overnight incubation, the DCs were further incubated in the absence or presence of 0.1 µg/ml CT for 24 h followed by cell collection for flow cytometric analysis.

### Statistical Analysis

Mann–Whitney *U* test was used to calculate statistical differences between experimental and control measurements. Comparisons involving multiple groups were performed using two-way ANOVA with Bonferroni’s multiple comparisons test (Prism software 7). In figures, *P*-values <0.05, <0.01, <0.001, and <0.0001 are represented by the symbols *, **, ***, and ****, respectively.

## Results

### Defective Humoral and Cellular Immune Responses in S100A4-Deficient Mice after Mucosal Immunization with OVA and CT

**Figure 1:**
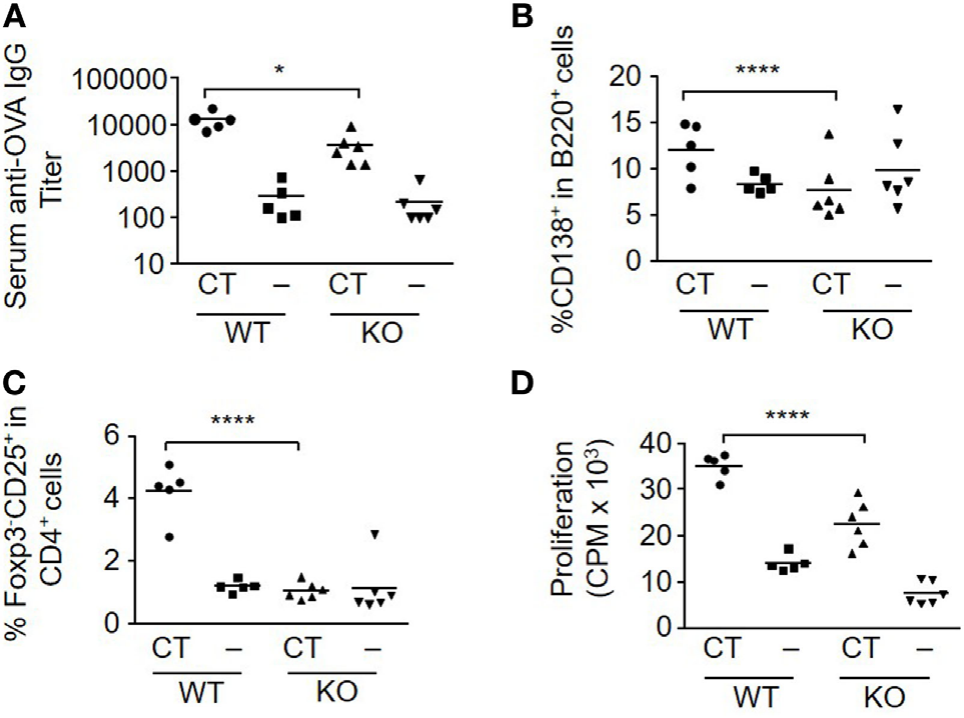
Loss of S100A4 impairs humoral and cellular immune responses after sublingual (s.l.) immunization with ovalbumin (OVA) and cholera toxin (CT). S100A4^+/+^ [wild-type (WT)] or S100A4^−/−^ [knockout (KO)] mice were treated s.l. with 200 µg OVA mixed with or without 5 µg CT twice at an interval of 10 days. Serum and spleens were collected 10 days after the last s.l. treatment. **(A)** Serum levels of anti-OVA IgG were measured by ELISA. **(B,C)** Percentage of splenic plasmablasts **(B)** and T effector cells **(C)** were examined by flow cytometry. **(D)** Splenocytes were incubated with 20 µg/ml OVA for 3 days followed by measurement of T cell proliferation by cellular [^3^H]-thymidine incorporation. Each symbol represents data from one individual mouse. *P*-values are derived from two-way ANOVA with Bonferroni’s multiple comparisons test (Prism software 7) comparing the WT and KO mice. *P < 0.05; ****P < 0.0001.

To evaluate the influence of S100A4 on the immune response to a model protein antigen after mucosal immunization, WT and S100A4^−/−^ mice were s.l. immunized twice with OVA alone or together with CT as an adjuvant. Most protein antigens are poorly immunogenic when administered mucosally without an effective mucosal adjuvant. This was confirmed for the s.l. immunization with OVA, which when given alone failed to stimulate more than a marginal immune response. However, when OVA was given with CT, a strong serum IgG anti-OVA antibody response was elicited in WT mice, which was approximately 10-fold higher than that obtained in the S100A4^−/−^ mice (Figure 1A; Figure S1 in Supplementary Material). The decreased antibody response in S100A4^−/−^ mice was associated with much reduced generation of splenic B220^+^CD138^+^ plasmablasts (Figure 1B) as well as reduced numbers of activated (CD69^+^) and migrated (CD103^+^) splenic B cells (data not shown).

T cell responses after CT-adjuvanted immunization were also strongly reduced in the S100A4^−/−^ mice. In the OVA plus CT-immunized WT mice, there was a marked increase in the number of T effector cells (Foxp3^−^CD25^+^CD4^+^) in response to immunization, which was absent in similarly immunized S100A4^−/−^ mice (Figure 1C). The peak T cell proliferative response was also significantly higher in WT mice than in S100A4^−/−^ mice after immunization with OVA plus CT (Figure 1D). Consistent with our previous studies, CT significantly inhibited the levels of regulatory T cell (Treg) (Foxp3^+^CD4^+^) cells in WT mice (23). However, no such CT-mediated effect was seen in S100A4^−/−^ mice, which also had higher baseline levels of Treg (data not shown).

### Lack of S100A4 Abrogates the Germinal Center Reaction

**Figure 2:**
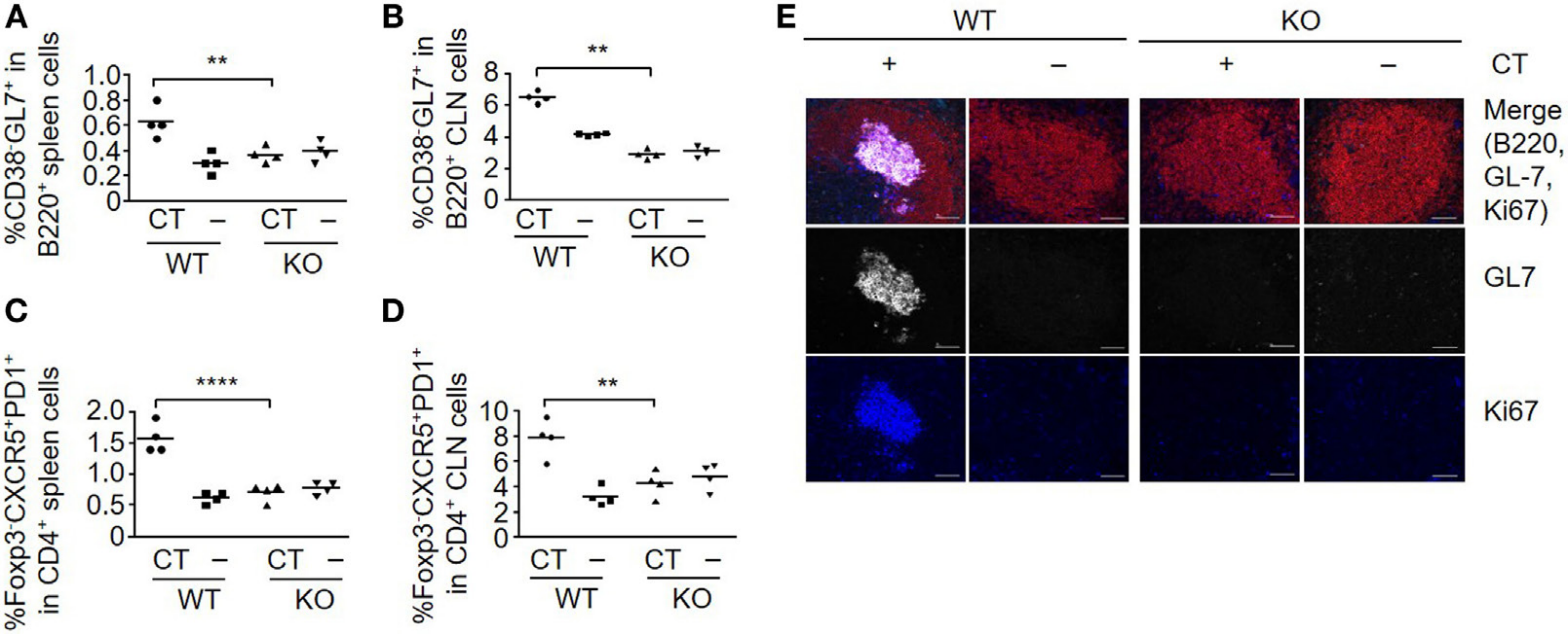
Loss of S100A4 abrogates germinal center responses. S100A4^+/+^ [wild-type (WT)] or S100A4^−/−^ [knockout (KO)] mice were sublingually (s.l.) treated with 200 µg ovalbumin mixed with or without 5 µg cholera toxin (CT) twice at an interval of 10 days. **(A–D)** Spleens **(A,C)** and cervical lymph nodes (CLN) **(B,D)** were collected 3 days after the last s.l. treatment. Frequencies of spleen cell compartments that characterize germinal center formation including GL7^+^ B cells **(A,B)** and T follicular helper cells **(C,D)** were analyzed by flow cytometry. **(E)** Spleen sections were analyzed by immunohistochemistry to visualize germinal center B cells. Red, B220; white, GL7; blue, Ki67. Upper panels, merged images. Scale bar, 50 µm. Each symbol represents data from one individual mouse **(A–D)**. Representative data were obtained from two similar experiments **(E)**. *P*-values are derived from two-way ANOVA with Bonferroni’s multiple comparisons test (Prism software 7) comparing the WT and KO mice. **P < 0.01; ****P < 0.0001.

The induction of humoral and cellular responses is associated with stimulation of germinal center formation. The B-cell subset that expresses GL7, but not CD38, which is defined as germinal center-localized cells (24, 25), was efficiently induced in both spleen (Figure 2A) and CLNs (Figure 2B) in WT, but not in S100A4^−/−^ mice after immunization with OVA plus CT. The immunized WT mice also produced T follicular helper (Tfh) cells (Foxp3^−^CXCR5^+^PD1^+^), which contribute to class-switch recombination, somatic hypermutation, and memory B-cell development in germinal centers (25, 26) in both spleens (Figure 2C) and CLNs (Figure 2D). In contrast, there was no Tfh cell response in either the spleens or CLNs of immunized S100A4^−/−^ mice. These observations were further supported by the immunohistochemical identification of splenic germinal centers in WT, but not in S100A4^−/−^ mice after immunization with OVA plus CT (Figure 2E).

### Engraftment with WT DCs Restores Mucosal Immunization Responses in S100A4^−/−^ Mice

Following immunization, immature DCs take up antigen and adjuvant, and then migrate into draining lymph nodes for subsequent stimulation of antigen-specific T cells. To examine the role of S100A4 in early-stage s.l. mucosal induction of adaptive immune responses, WT and S100A4^−/−^ mice were given a single administration of OVA with or without CT, and 2 days later CLNs, which are the primary draining lymph nodes after s.l. immunization, were collected and analyzed for markers defining the induction of adaptive immune responses. As shown in Figure S2 in Supplementary Material, in WT mice, CT-adjuvanted immunization efficiently expanded the pools of CD11c^+^ DCs expressing the co-stimulatory molecule CD86 as well as of CD69^+^Foxp3^−^ activated T cells, whereas in contrast, these CT-induced cellular responses were completely absent in S100A4^−/−^ mice.

**Figure 3:**
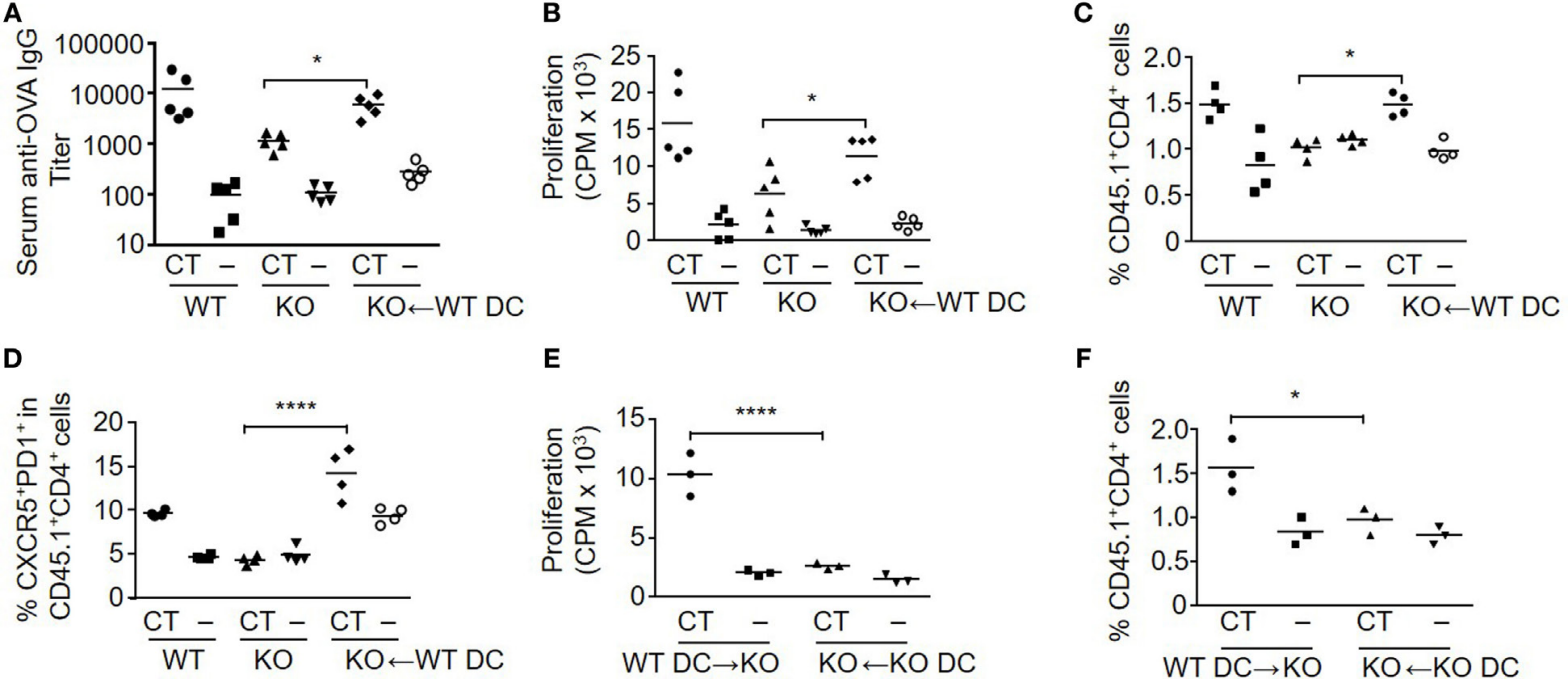
Engraftment of S100A4^−/−^ [knockout (KO)] mice with S100A4^+/+^ [wild-type (WT)] dendritic cells (DCs) restores adaptive immune responses. **(A–D)** Freshly isolated CD11c^+^ DC from WT mice were adoptively intravenously transferred into a subset of the KO mice. All the mice in the immunization experiment also received CD45.1^+^ OT-IIxLy5.1 T cells. Mice were then treated as described in Figure 1. Serum levels of anti-ovalbumin (OVA) IgG were measured by ELISA **(A)**. Splenic T cell proliferation *in vitro* was measured by cellular [^3^H] incorporation **(B)**. The percentage of splenic OT-II T cells representing OT-II T cell expansion **(C)** and percentage of T follicular helper cells among OT-II T cells **(D)** were analyzed by flow cytometry. **(E,F)** In a separate experiment, similar manipulations as explained earlier were carried out. Splenic T cell proliferation **(E)** and percentage of splenic OT-II T cells **(F)** from S100A4^−/−^ mice engrafted with WT or S100A4^−/−^ DCs were recorded. Each symbol represents data from one mouse. *P*-values are derived from two-way ANOVA with Bonferroni’s multiple comparisons test (Prism software 7) comparing the intact KO mice and KO mice that had received WT DC **(A–D)**, or comparing KO mice that had received WT DCs and KO mice that had received KO DCs **(E,F)**. *P < 0.05; ****P < 0.0001.

To further assess the role of S100A4 in DCs for the induction of antigen-specific T cell activation and antibody responses after mucosal immunization, we compared the immune responses of WT mice, S100A4^−/−^ mice, and S100A4^−/−^ mice who had adoptively received WT DCs one day before the first of the standard 2-dose s.l. immunization with OVA. In addition, at the time of DC transfer, all the mice had adoptively received OT-II T cells whose expression of CD45.1 can be used to facilitate analysis of antigen-specific T cell expansion *in vivo*. Consistent with the results in Figure 1, S100A4^−/−^ mice had impaired humoral and cellular immune responses compared to WT mice. In contrast, both humoral and cellular responses were largely restored to WT levels in S100A4^−/−^ mice that had received WT DCs before immunization. In the immunized S100A4^−/−^ engrafted mice, the antigen-specific antibody (Figure 3A) and antigen re-encounter-induced T cell *in vitro* proliferation (Figure 3B) responses were closely similar to those of immunized WT mice. The CT/antigen-induced expansion of Foxp3^−^CD25^+^CD4^+^ effector T cells and downregulation of splenic Treg observed in WT mice were also restored in S100A4^−/−^ mice that had been engrafted with WT DCs (data not shown). The *in vivo* expansion of antigen-specific OT-II T cells was also restored to WT levels in S100A4^−/−^ mice following engraftment with WT DCs (Figure 3C), and the engraftment also restored the generation of Tfh cells that are associated with germinal center responses (Figure 3D). Notably, engraftment of S100A4^−/−^ mice with S100A4^−/−^ DCs neither *in vitro* (Figure 3E) nor *in vivo* (Figure 3F) restored T cell expansion, supporting that the rescue of T cell responses observed in S100A4^−/−^ mice engrafted with WT DCs cannot be explained by a non-specific increase in DC numbers in these animals.

### CT Adjuvant-Stimulated DC Activation Requires S100A4 for Mature IL-1β Production and Antigen Presentation

**Figure 4:**
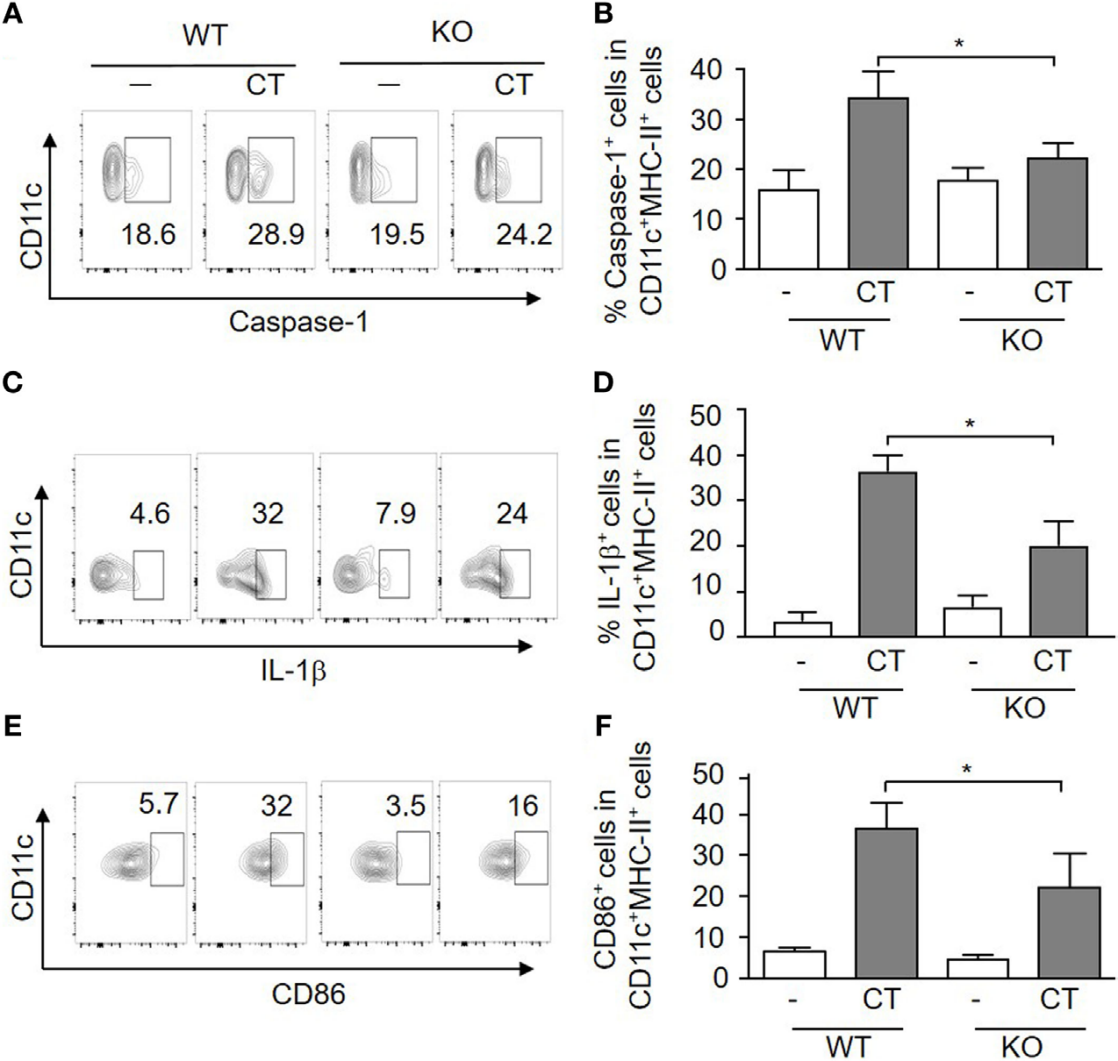
Loss of S100A4 affects cholera toxin (CT)-induced dendritic cell (DC) activation. Bone marrow-derived DCs from S100A4^+/+^ [wild-type (WT)] or S100A4^–/–^ [knockout (KO)] mice were treated overnight with ovalbumin in the presence or absence of CT. Cell surface expression of CD11c together with caspase-1 **(A,B)**, intracellular IL-1β **(C,D)**, and CD86 **(E,F)** was examined by flow cytometry. Numbers beside gated regions indicate percent cells in each **(A,C,E)**. The fold change in cell number following CT-mediated immunization over control is expressed as the mean ± SD for three independent experiments **(B,D,F)**. *P*-values are derived from two-way ANOVA with Bonferroni’s multiple comparisons test (Prism software 7) comparing DCs from WT and KO mice. *P < 0.05.

The adjuvant activity of CT has been linked to inflammasome activation, which promotes IL-1 activation and secretion in APCs (22), and S100A4 has been implicated in the regulation of the IL-1β-mediated matrix metalloproteinase-13 production (27). Therefore, we evaluated if inflammasome-driven IL-1β production and caspase-1 activation in APCs was altered in S100A4^−/−^ mice by comparing the expression of these molecules along with the co-stimulatory molecules CD86 and CD80 in WT and S100A4^−/−^ DCs. The results are shown in Figure 4 and demonstrate that CT-treated WT BMDCs exhibited increased frequencies of caspase-1 (Figures 4A,B), pre-IL-1β (Figures 4C,D), CD86 (Figures 4E,F), and CD80 (data not shown) positive cells as compared to S100A4^−/−^ BMDCs. The histogram profiles of these flow cytometric analyses are provided for better visual comparisons (Figures S3A–C in Supplementary Material). For CD86 expression, as there is no distinct positive population revealed, we also measured the mean fluorescence intensity (Figure S3D in Supplementary Material), which is in agreement with the contour and histogram comparisons (Figures 4E,F; Figure S3C in Supplementary Material). Our data suggest that S100A4 may impact caspase-1 activation more substantially compared with the production of the latent pre-IL-1β, as S100A4^−/−^ BMDCs were able to upregulate IL-1β upon activation moderately in contrast to an almost complete lack of caspase-1 upregulation (Figures 4B,D). The implication of this observation is that the cleaved, bioactive IL-1β may be more tightly regulated by S100A4. Furthermore, these DC function-associated analyses at the protein level were corroborated by observations of similar reductions in mRNA expression for CD80, CD86, IL-1β, and IL-6 in S100A4^−/−^ DCs compared to WT DCs in response to treatment with CT *in vitro* (Figure S4 in Supplementary Material).

**Figure 5:**
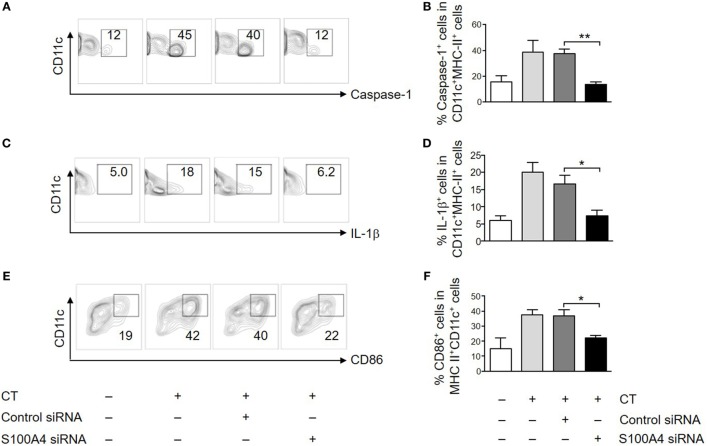
Silencing S100A4 inhibits cholera toxin (CT)-induced dendritic cell (DC) activation. Bone marrow-derived DCs were transfected with S100A4 siRNA or control siRNA. The cells were next incubated for another overnight with ovalbumin in the presence or absence of CT. Cell surface expression of CD11c together with caspase-1 **(A,B)**, intracellular IL-1β **(C,D)**, and CD86 **(E,F)** was examined by flow cytometry. Numbers beside gated regions indicate percent cells in each **(A,C,E)**. The fold change in cell number following CT-mediated immunization over control is expressed as mean ± SEM for three independent experiments **(B,D,F)**. *P < 0.05, **P < 0.01 by Mann–Whitney *U* test comparing the groups with S100A4-targeting siRNA and the control siRNA.

To further evaluate the importance of S100A4 in APC activation by CT, we also tested if *in vitro* transfection of DCs with an S100A4-targeting siRNA could specifically inhibit CT-induced surface expression of CD86 and intracellular expression of caspase-1 and IL-1β. In CT-treated DCs transfected with control siRNA, there was no difference in expression of any of these molecules as compared to CT-treated non-transfected WT DCs (Figure 5; Figure S5 in Supplementary Material). In contrast, the expression of CD86, caspase-1 and IL-1β was consistently reduced in DCs transfected with the S100A4-specific siRNA (Figure 5; Figure S5 in Supplementary Material). These findings in WT DCs transfected with S100A4-specific siRNA strongly support the finding in S100A4^−/−^ DCs that S100A4 is required for CT-induced adjuvant activation of DCs.

**Figure 6:**
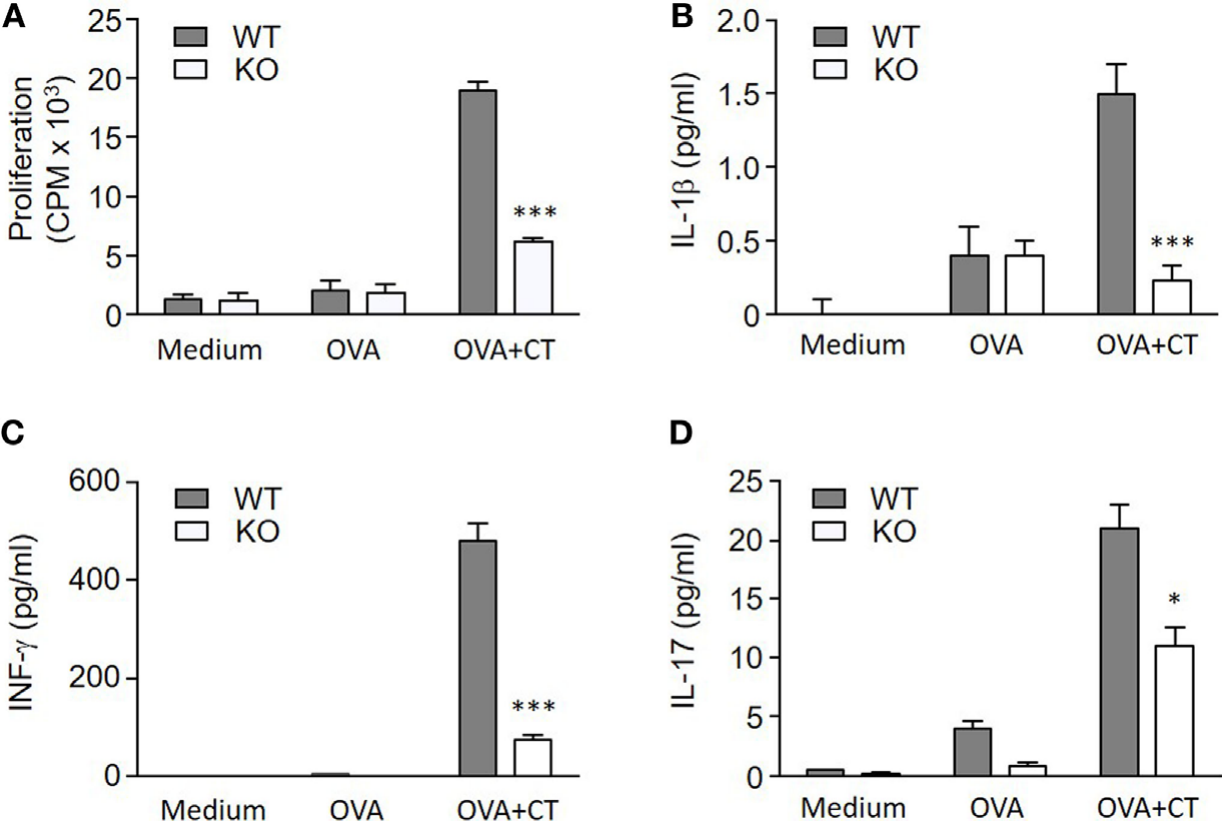
S100A4^−/−^ dendritic cells (DCs) fail to efficiently activate T cells. **(A)** OT-IIxLy5.1 T cell proliferation was measured by cellular [^3^H] thymidine incorporation following coculture with S100A4^+/+^ [wild-type (WT)] or S100A4^−/−^ [knockout (KO)] bone marrow-derived DCs that had been pulsed with medium alone, ovalbumin (OVA), or OVA mixed with cholera toxin (CT) as indicated. **(B–D)** Coculture supernatants were collected for measurement of IL-1β **(B)**, IFN-γ **(C)**, and IL-17 **(D)** by the Meso Scale Discovery assay. Data are plotted as the mean ± SEM for three mice. *P < 0.05, ***P < 0.001 by Mann–Whitney *U* test comparing the cocultures containing WT DCs and knockout (KO) DCs that had been treated with CT and OVA.

To determine if the lack of S100A4 in DCs affected the induction of antigen-specific T cell responses, OVA and CT-treated WT or S100A4^−/−^ BMDCs were cocultured with CD4^+^ T cells from OT-IIxLy5.1 mice. The stimulation of OVA-specific T cell proliferation by OVA plus CT-treated S100A4^−/−^ DCs was strongly reduced compared to the stimulation by similarly treated WT DCs (Figure 6A). Cytokine analyses also revealed that the levels of CT-stimulated IL-1β, IFN-γ, and IL-17 in medium derived from the cocultured S100A4^−/−^ DCs and CD4^+^ T cells were reduced as compared to those from WT DC/T cell cocultures (Figures 6B–D).

## Discussion

In this study, we demonstrate that S100A4 is important for efficient induction of adaptive immune responses after s.l. immunization with the model protein antigen OVA given together with the strong mucosal adjuvant CT. Both humoral and cellular immune responses were markedly compromised in S100A4-deficient mice compared to WT mice, and S100A4 was found to be critically important for germinal center formation and Tfh cell development in response to immunization. Our findings further provide compelling evidence that the impaired humoral and cellular immune responses in S100A4^−/−^ mice following mucosal immunization can largely, if not exclusively, be attributed to ineffective antigen presentation due to poor responsiveness of S100A4^−/−^ APCs to the adjuvant action of CT. *In vitro*, DCs from S100A4^−/−^ mice failed to activate antigen-specific T cells after exposure to OVA plus CT, which could be related to their defective responsiveness to adjuvant stimulation by CT. The requirement for S100A4 in APC function was further supported by engraftment studies demonstrating that the transfer of WT DCs but not S100A4^−/−^ DCs to S100A4-deficient mice fully restored the immune responsiveness of the recipients to subsequent immunization with OVA plus CT. Our work identifies an important role for S100A4 in the adjuvant action of CT (and we assume also of other adjuvants) and adds to current knowledge about the role of S100A4 in the development of adaptive immune responses to immunization by demonstrating its critical importance in APCs for effective antigen presentation.

The importance of the germinal center reaction and Tfh cells for efficient humoral immune responses has recently attracted great attention (25, 26, 28). Our data demonstrate that the loss of S100A4 completely abrogated the formation of germinal centers and the development of Tfh cells in response to s.l. immunization with OVA plus CT as compared to WT mice. Interestingly, despite the almost complete lack of germinal center reaction in S100A4-deficient mice, these mice retained modest antigen-specific antibody production and T cell proliferation, which may reflect the existence of germinal center-independent immune responses (29). However, as germinal centers are critical foci where affinity maturation and memory responses of B cells are induced, it may be assumed that the adaptive immune responses in the absence of S100A4 are compromised not only quantitatively but also qualitatively. Future studies assessing the requirement for S100A4 in affinity maturation and memory responses after immunization will help to delineate the roles of S100A4 in immune regulation.

The WT and S100A4^−/−^ mice compared in this study were not littermates, which could be a limitation in case possible differences in microbiota would have influenced the comparisons. However, two sets of results practically rule out the possibility that any such difference rather than the presence or absence of S100A4 in APCs could explain the defective immune responsiveness of the S100A4^−/−^ mice. Thus, our finding that transfer of WT DCs to S100A4^−/−^ mice before immunization practically completely restored the immunological responsiveness provides strong evidence for the specific role of S100A4 in the induction of a normal immune response, as does our finding that transfection of DCs from WT mice with S100A4-specific siRNA but not mock-siRNA resulted in impaired expression of the immunostimulatory molecules CD86, IL-1β and caspase-1 in response to CT treatment *in vitro*.

The adjuvant mechanism of CT, whose enterotoxic activity is closely linked to its ADP-ribosylating activity that mediates increased cAMP formation and chloride secretion in enterocytes, has been extensively studied (30). CT, as well as the analogous heat-labile enterotoxin from *Escherichia coli* (LT), and their relatively non-toxic adjuvant-active derivatives mmCT and dmLT all induce enhanced T cell proliferative and cytokine (predominantly IL-17) responses in DCs and other APCs through mechanisms involving cAMP/protein kinase A-dependant inflammasome activation and IL-1 production (22, 31). Both CT and its non-toxic B subunit are also known to promote influx of calcium into cells (32). Calcium signaling is required for the maturation of human DCs and for promoting immune responses (33, 34). Studies are in progress to define more precisely the molecular pathways in DCs and other APCs linking S100A4-mediated calcium signaling to cAMP/PKA-dependent IL-1 production and antigen presentation.

In conclusion, this study identifies S100A4 as a critical regulator for CT adjuvant-mediated induction of both cellular and humoral adaptive immune responses after mucosal immunization. Our data demonstrate that a lack of S100A4 leads to compromised antigen presentation, resulting in impaired helper and effector T cell activation and a marked suppression of the development of Tfh cells and germinal centers. This study was limited to investigating the role of S100A4 for CT-adjuvanted antigen presentation and adaptive immune responses, but it seems likely that other adjuvants and types of immune responses may also be influenced in similar ways by S100A4 and calcium signaling. In support of the latter, we have previously described that S100A4-deficient mice had reduced antibody and T cell responses after parenteral immunization with OVA adjuvanted with alum associated with defective T cell antigen presentation *in vitro* by S100A4^−/−^ DCs (15). Work is in progress to further define to which extent the important role of S100A4 demonstrated for the adjuvant activity of CT may apply also to other antigens, adjuvants and immunization routes.

## Ethics Statement

This study was carried out in accordance with the recommendations of the Ethical Committee for Animal Experimentation in Gothenburg, Sweden. The protocol was approved by the Ethical Committee for Animal Experimentation in Gothenburg, Sweden.

## Author Contributions

J-BS, JH, and ZX conceived and designed the study. J-BS and ZX performed the experiments and analyzed the data with the help from ML, MT, and YF. AB provided critical research tools including S100A4-deficient mice and contributed intellectual input. JH supervised the study. J-BS, JH, and ZX wrote the manuscript. All the authors read and approved the final version of the manuscript.

## Conflict of Interest Statement

The authors declare that the research was conducted in the absence of any commercial or financial relationships that could be construed as a potential conflict of interest.

## Abbreviations

BM, bone marrow; CLN, cervical lymph node; CT, cholera toxin; DC, dendritic cell; Foxp3, the forkhead-winged helix family transcription factor; Teff, effector T cell; Tfh, T follicular helper cell; Treg, regulatory T cell; WT, wild-type; KO, knockout.
