# Supplementary material for: Deficiency in Calcium-Binding Protein S100A4 Impairs the Adjuvant Action of Cholera Toxin
Source: Front Immunol. 2017 Sep 11;8:1119. doi: 10.3389/fimmu.2017.01119 (PMC5600718; doi:10.3389/fimmu.2017.01119)
Supplement: Supplementary file 1 [file Image_1.pdf]

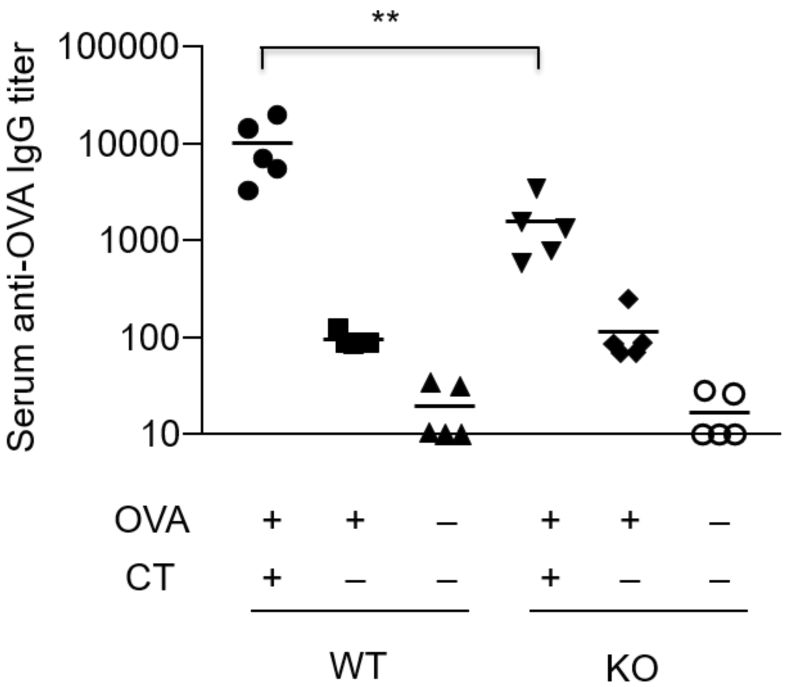

Figure S1. Impairment of humoral immune response as a result of deficiency in S100A4 is seen after immunization with ovalbumin (OVA) plus cholera toxin (CT) but not with OVA alone. S100A4$^{+/+}$ [wild-type (WT)] or S100A4$^{-/-}$ [knockout (KO)] mice were sublingually (s.l.) treated with or without 200 μg OVA together with or without 5 μg CT twice with an interval of 10 days as indicated. "–" denotes administration with PBS as control. Sera were collected 10 days after the last immunization and the levels of anti-OVA IgG were measured by ELISA. *P*-values are derived from 2-way ANOVA with Bonferroni's multiple comparisons test (Prism software 7) comparing the WT and KO mice. $^{**}P < 0.01$.

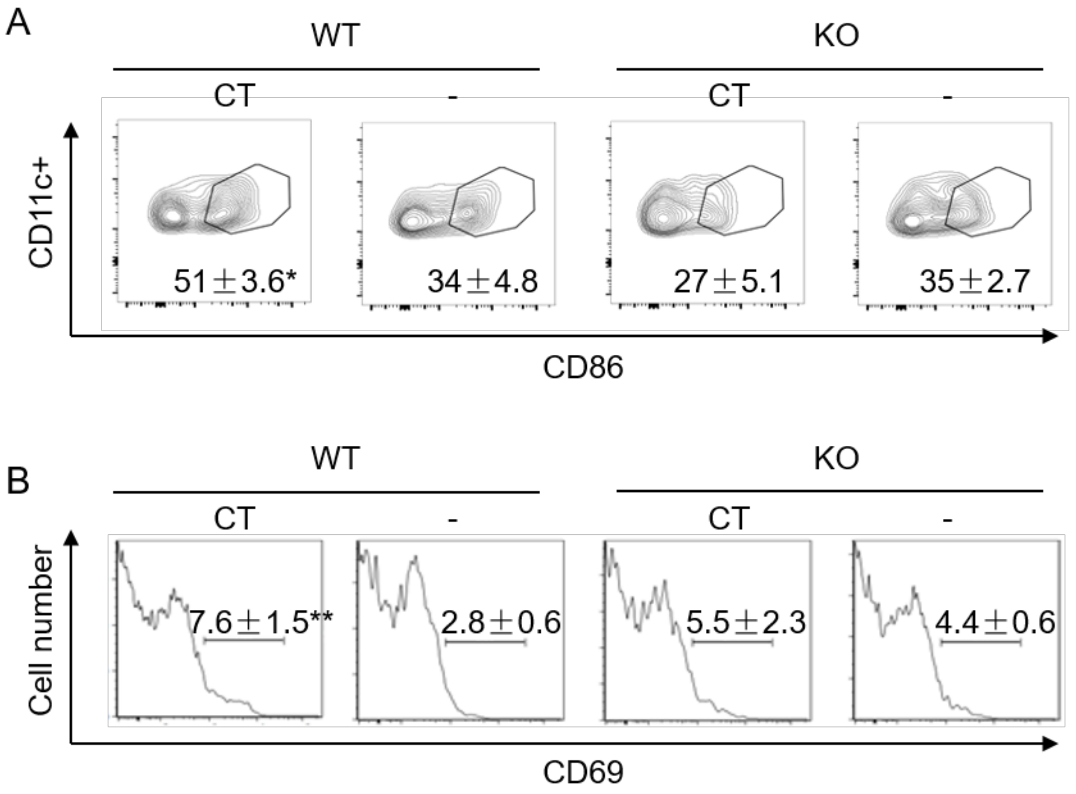

Figure S2. S100A4 is required for initiation of adaptive immune responses after sublingual (s.l.) immunization with ovalbumin (OVA) and cholera toxin (CT). S100A4$^{+/+}$ [wild-type (WT)] or S100A4$^{-/-}$ [knockout (KO)] mice were treated s.l. with 200 μg OVA mixed with or without 5 μg CT once. Two days after immunization, cervical lymphnodes were collected and analyzed by flow cytometry to examine the frequency of CD86^{+} DCs within the CD11c^{+} DC population **(A)**, and CD69^{+} effector T cells within the Foxp3^{-}CD4^{+} cell population **(B)**. Numbers beside gated regions or above the horizontal bars indicate percent cells in that compartment. Data are expressed as mean ± SD. *, $P < 0.05$; **, $P < 0.01$, for indicating the statistical significance between the groups with or without CT treatment for the WT mice.

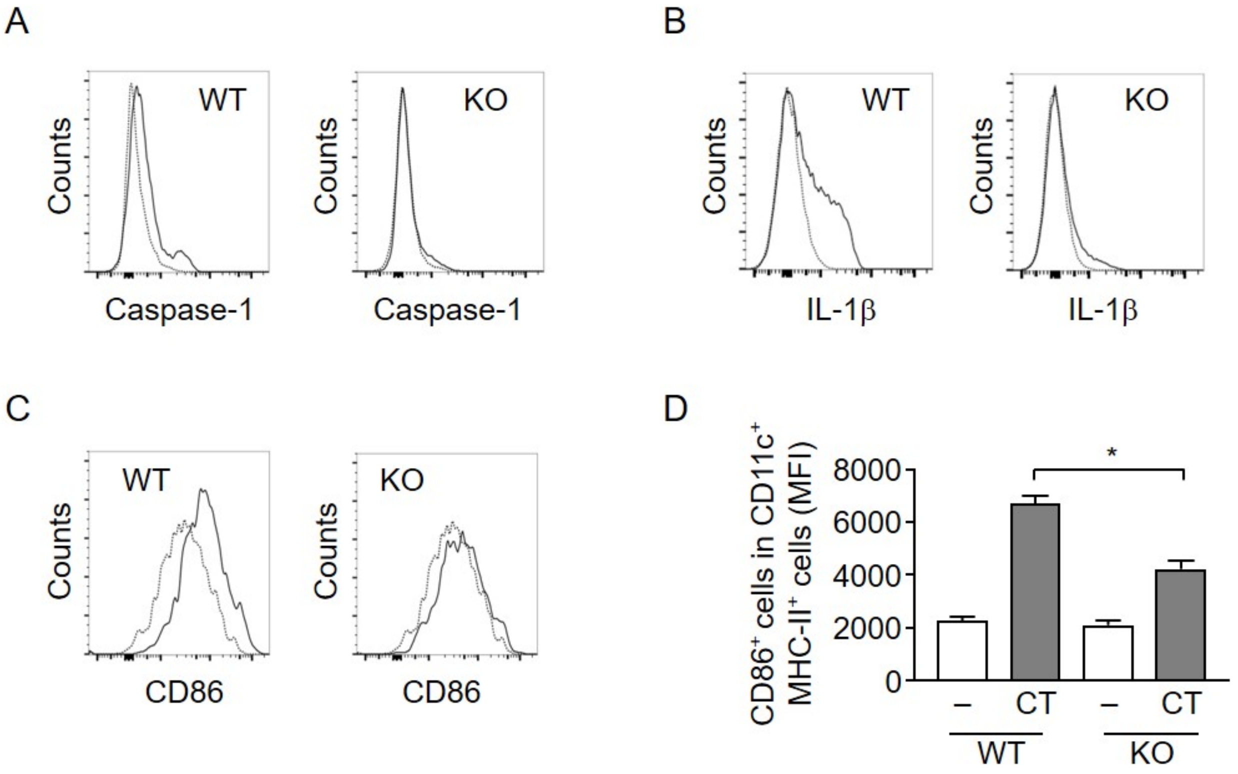

Figure S3. Display of the same raw flow cytometry data shown in Figure 4 in histograms and as mean fluorescence intensity (MFI). **(A to C)** Bone marrow-derived dendritic cells (DCs) from S100A4$^{+/+}$ [wild-type (WT)] or S100A4$^{-/-}$ [knockout (KO)] mice were treated overnight with ovalbumin in the presence (solid lines) or absence (dotted lines) of cholera toxin. CD11c^{+} dendritic cells (DC) were analyzed for the expression of caspase-1 **(A)**, intracellular latent IL-1β **(B)**, and CD86 **(C)**. The MFI values of the measurements shown in **(C)** were also plotted as mean ± SEM for three independent experiments **(D)**. *P*-values are derived from 2-way ANOVA with Bonferroni's multiple comparisons test (Prism software 7) comparing DCs from WT and KO mice **(D)**. *$P < 0.05$.

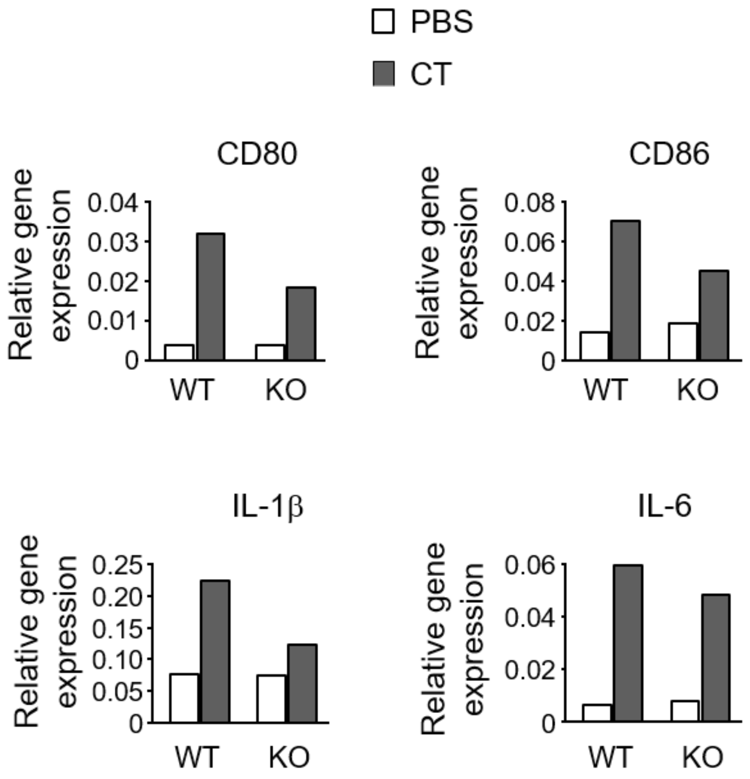

Figure S4. Impaired induction of mRNA messages for various immune regulatory molecules in response to cholera toxin (CT) treatment by S100A4$^{-/-}$ [knockout (KO)] dendritic cells (DC) as compared to S100A4$^{+/+}$ [wild-type (WT)] DCs *in vitro*. Bone marrow-derived DCs were incubated with medium alone (empty) or 1 μg/ml CT (solid) for 24 h followed by measurement of mRNA expression by PCR array. Data show results from one of two experiments with similar results.

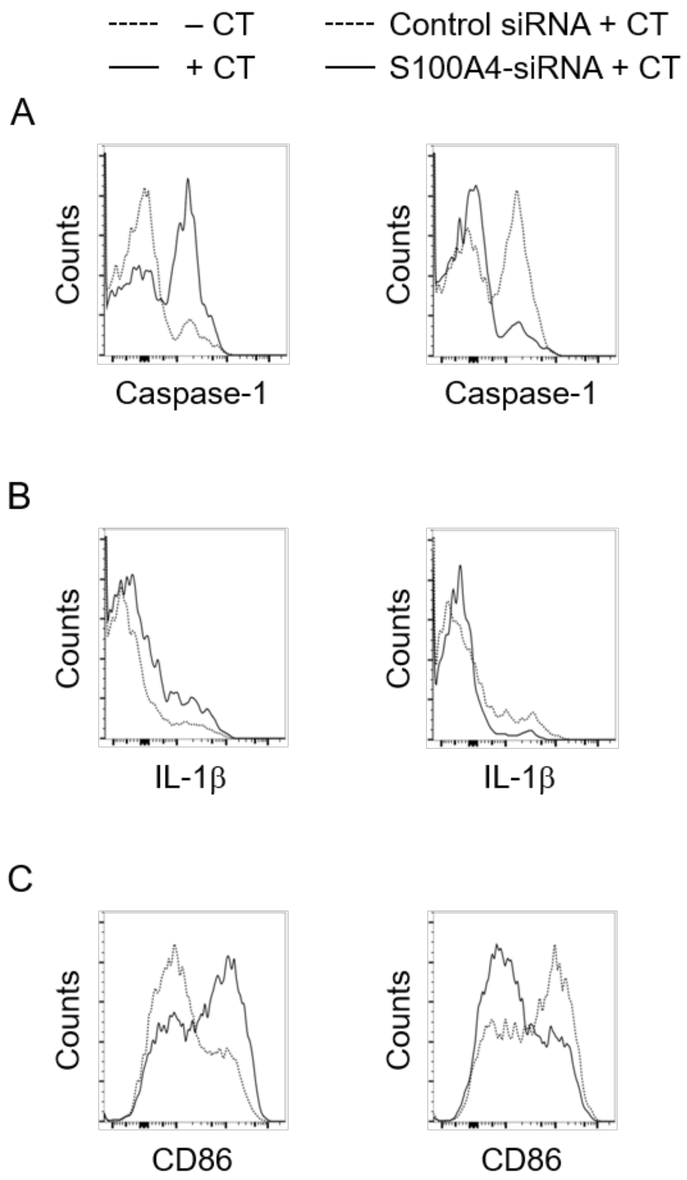

Figure S5. Display of the same raw flow cytometry data shown in Figure 5 in histograms. Bone marrow-derived dendritic cells (DCs) were incubated in the absence or presence of CT. Some of the cells were transfected with S100A4-targeting siRNA or control siRNA prior to CT treatment. $CD11c^+$ DCs were analyzed for the expression of caspase-1 **(A)**, latent intracellular IL-1β **(B)**, and CD86 **(C)**.
